# Metabolomic Profile of Young Adults Born Preterm

**DOI:** 10.3390/metabo11100697

**Published:** 2021-10-12

**Authors:** Serafina Perrone, Simona Negro, Elisa Laschi, Marco Calderisi, Maurizio Giordano, Giuseppe De Bernardo, Gianni Parigi, Anna Laura Toni, Susanna Esposito, Giuseppe Buonocore

**Affiliations:** 1 Department of Medicine and Surgery, University of Parma, 43126 Parma, Italy; susannamariaroberta.esposito@unipr.it; 2 Maternity and Childhood Department, Neonatal Intensive Care, University Hospital of Siena, 53100 Siena, Italy; simonanegro.84@gmail.com; 3 Department of Molecular and Developmental Medicine, University of Siena, 53100 Siena, Italy; elisa.laschi87@gmail.com (E.L.); gianni.parigi@yahoo.it (G.P.); annalauratoni@gmail.com (A.L.T.); giuseppe.buonocore@unisi.it (G.B.); 4 Kode Solution srl, 56125 Pisa, Italy; m.calderisi@kode-solutions.net; 5 Department of Clinical Medicine and Surgery, Federico II University, 80131 Naples, Italy; giordan_maurizio@libero.it; 6 Department of Woman and Child, Ospedale Buon Consiglio Fatebenefratelli, 80123 Naples, Italy; pinodebtin@gmail.com

**Keywords:** preterm newborn, NMR spectroscopy, urine samples

## Abstract

Prematurity is a risk factor for the development of chronic adult diseases. Metabolomics can correlate the biochemical changes to a determined phenotype, obtaining real information about the state of health of a subject at that precise moment. Significative differences in the metabolomic profile of preterm newborns compared to those born at term have been already identified at birth. An observational case–control study was performed at the University Hospital of Siena. The aim was to evaluate and compare the metabolomic profiles of young adults born preterm to those born at term. Urinary samples were collected from 67 young adults (18–23 years old) born preterm (mean gestational age of 30 weeks, *n* = 49), and at term of pregnancy (mean gestational age of 38 weeks, *n* = 18). The urinary spectra of young adults born preterm was different from those born at term and resembled what was previously described at birth. The Random Forest algorithm gave the best classification (accuracy 82%) and indicated the following metabolites as responsible for the classification: citrate, CH2 creatinine, fumarate and hippurate. Urine spectra are promising tools for the early identification of neonates at risk of disease in adulthood and may provide insight into the pathogenesis and effects of fetal programming and infants’ outcomes.

## 1. Introduction

Fetal and extrauterine life represents a continuum, during which the growth and development of the human being are influenced by genetic, environmental and social factors. Numerous studies have identified prematurity as a risk factor for the development of chronic adult diseases such as obesity, insulin resistance [1,2] and hypertension [3,4]. Recent evidence documents the fetal, rather than postnatal, origin of some chronic adult diseases. It is likely that fetal reprogramming occurs when the normal pattern of fetal development is disrupted by an abnormal stimulus or an “insult” during intrauterine life, which leads to adaptations by the fetus to allow for its survival, but could ultimately result in permanent structural and physiological changes with long-term consequences in adulthood. Early in utero life is vulnerable to perturbation, and compelling evidence indicates that the fetal period of development is extremely sensitive to environmental cues. Insufficient fetal substrates determine permanent structural and physiological changes, leading to long-lasting consequences in postnatal life [5,6]. Many experimental studies have been conducted to explain the phenotypic consequences of fetal–placental perturbations that predispose individuals to the genesis of metabolic syndrome in adulthood. Metabolomics is an emerging omics science, considered today as the key for personalized medicine, able to correlate the biochemical changes (characterizing the organism of the human being, exposed to multiple intrinsic and extrinsic stresses) with a determined phenotype, and obtaining real information about the state of health of a subject at that precise moment [7]. Metabolomics has already identified significant differences at birth in the profile of preterm newborns compared to those born at term. Gracie et al. studied the importance and value of omics technologies and integrated them precisely for the study of preterm newborns [8]. Distinct metabolomic profiles were identified in infants born at different gestational ages, both in term and in preterm newborns [9], and in fetal growth-restricted infants [10]. However, very few studies have extended the follow-up of preterm infants into adult life [11,12,13]. The aim of our work is to identify and to compare the metabolomic profile of young adults born preterm to term controls, testing the hypothesis that metabolic profile in adulthood differs according to gestational age and resembles that of birth.

## 2. Results

**Figure 1:**
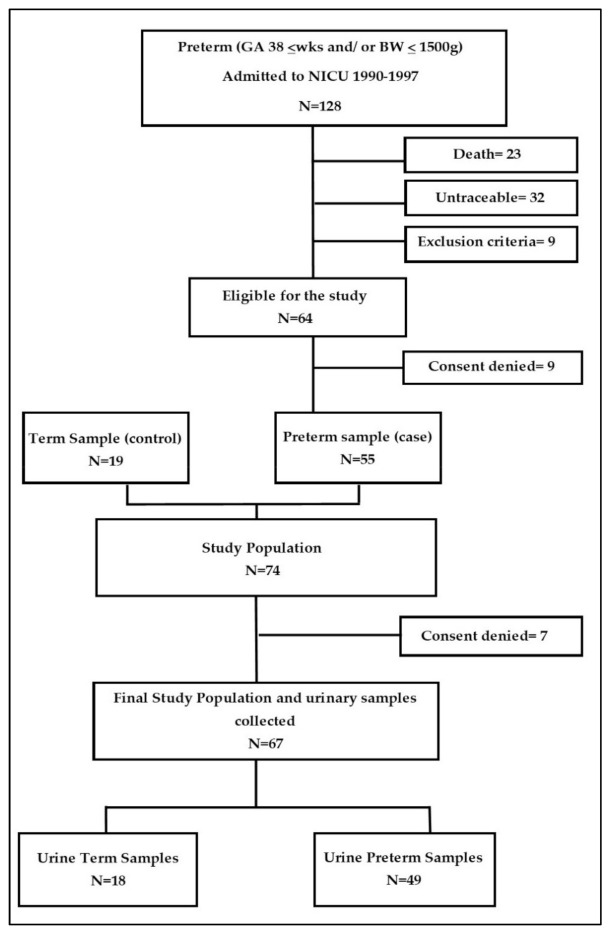
Participant flow chart; GA: gestational age; BW: body weight.

One hundred and twenty-eight preterm newborns met the inclusion criteria. Among them, 23 were deceased at the time of enrolment and 32 were untraceable through the available contact details. Nine were ineligible according to the exclusion criteria and 15 denied consent to participate in the study. Nineteen young adults born at term in the same study period (years 1990–1997) were selected as controls. One of them denied consent while the study was underway (Figure 1).

Therefore, the final study population consisted of 67 young adults: 49 born preterm (18 females and 31 males; gestational age: 30.25 ± 2.7 weeks; birth weight: 1131.91 ± 118.15, current age: 21 ± 2.4 years) and 18 born at term (6 females and 12 males; gestational age: 38.5 ± 1.4 weeks; birth weight: 3120.43 ± 261.02; current age: 20.9 ± 2.5). For the clinical characteristics of the enrolled population, see Table 1.

**Figure 2:**
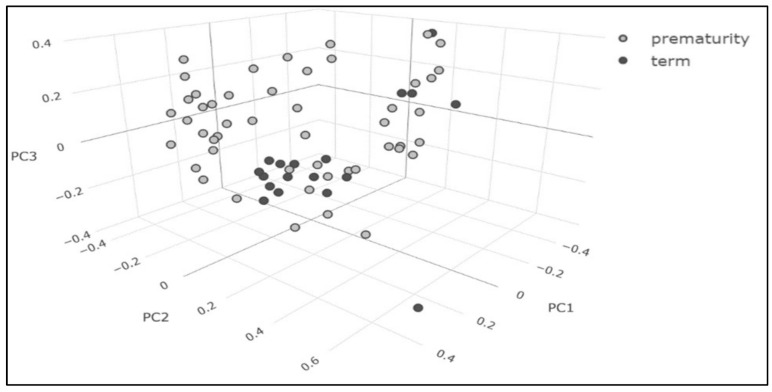
PCA score plot of the first three principal components; the two classes of patients, “preterm” and “term”, are represented in light gray and black points, respectively; PC: principal component.

Multivariate (chemometric) analysis allowed us to highlight differences in the urine metabolomic profile between young adults born preterm and young adults born at term. A non-supervised Principal Component Analysis (PCA) technique was performed to find clusters within the data set. The PCA did not show a clear difference between “preterm” and “term” clusters (Figure 2).

Therefore, the next supervised step by means of a supervised technique was required. Firstly, classification tasks were performed using nuclear magnetic resonance spectral data as the input for the classification models. A leave-one-out cross-validation technique was used as a resampling method to estimate the models’ performance. The models, nevertheless, were not yet very discriminative (the accuracy, i.e., the percentage of patients correctly classified by the predictive algorithm, was about 70%, Table 2).

**Figure 3:**
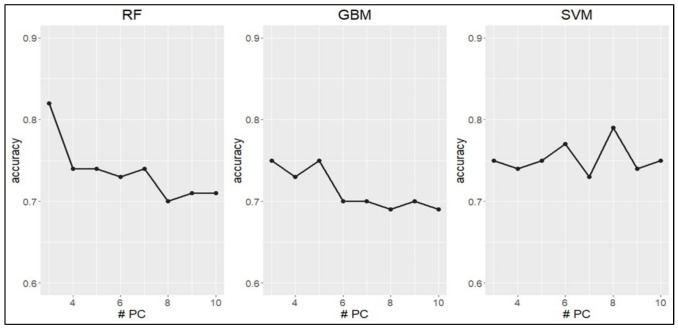
Accuracy of the models, estimated through the use of 3 to 10 PC as input data. RF: Random Forest; GBM: gradient boosting machine; SVM: support vector machine; PC: principal component.

Therefore, classification tasks were performed using principal components as input data. The models’ performances were estimated using 3 to 10 principal components to select the number of principal components with the best overall fit for each model (Figure 3).

**Figure 4:**
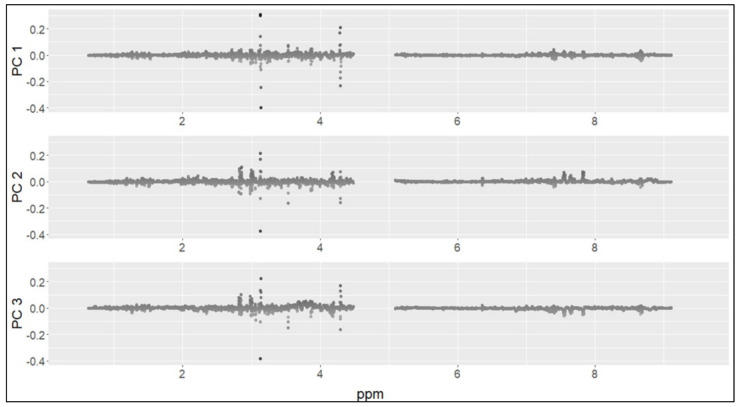
Values of the loadings of the first three principal components; the graphs illustrate which metabolite spectra (ppm) were most responsible for the “variance” in each of the three main components (i.e., the metabolite that has the higher absolute values).

The best classification result was obtained using the Random Forest (RF) model and the first three principal components as variables (accuracy ~82%). Moreover, to understand the contribution of each of the aforementioned components to the classification, an “importance” measure was computed through the RF algorithm. In order to identify the discriminating metabolites between preterm and term groups, the first three main components were then analyzed. The values of the loadings for each component were reported to understand how the metabolites contribute to each of the principal components (Figure 4).

**Figure 5:**
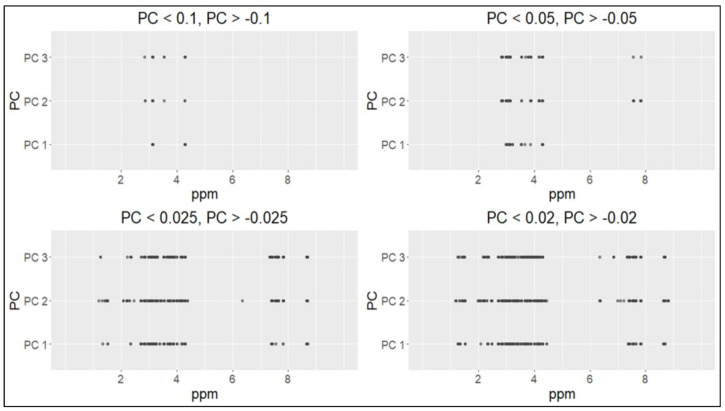
Different thresholds applied to the loadings of the first three principal components.

Positive values of the loadings indicate that a variable and a principal component are positively correlated; negative values indicate a negative correlation. Large (either positive or negative) values of the loadings indicate that a variable has a strong effect on that principal component. Some thresholds were set to select the variables with the highest absolute loading values (Figure 5).

**Figure 6:**
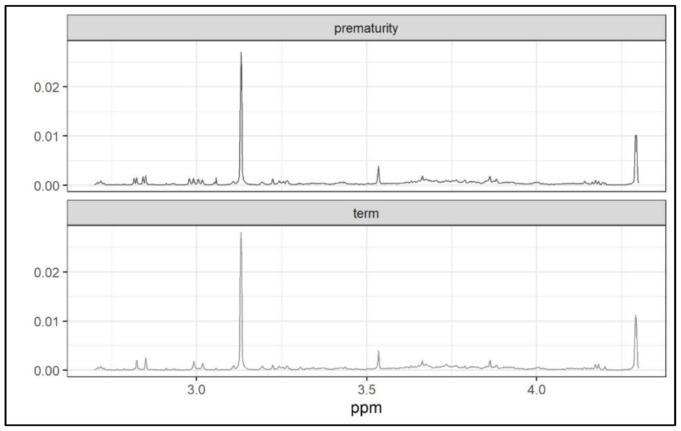
Comparison of mean spectra from each the two groups.

**Figure 7:**
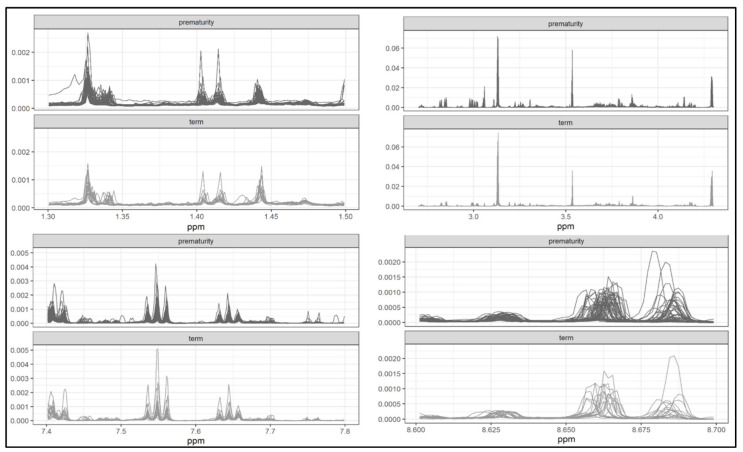
Spectra of the most important signals in the two groups.

The threshold values 0.1 and 0.05 were too high (only a few metabolites were selected for these values). For the threshold values 0.025 and 0.02, four ranges were identified in the ^1^H-NMR (proton nuclear magnetic resonance spectroscopy) spectrum: 1.3–1.5 ppm, 2.7–4.3 ppm, 7.4–7.8 ppm and 8.6–8.7 ppm (Figure 6 and Figure 7).

The most significant signals, which distinguished the metabolome of preterm from that of term newborns, came from the following metabolites: citrate (3.13 ppm), CH2 creatinine (4.28 ppm), fumarate (6.8 ppm) and hippurate (7.6–7.8 ppm).

## 3. Discussion

The main finding of this research was that the urine metabolomic profile of adults born preterm significantly differed from the metabolic profile of adults born at term. With the unlabeled metabolomics approach, we were able to identify the significant spectra, which differentiated the two young adult populations (preterm vs. term). In particular, the involved metabolomic cycles most related to the characterizing metabolites found in the group of preterm (citrate, CH3 creatinine, CH2 creatinine, fumarate and hippurate) were tyrosine metabolism, tryptophan and phenylalanine biosynthesis, the urea cycle and arginine and proline metabolisms. Interestingly, these metabolomic patterns were the same as those found and described by Atzori L et al. in preterm newborns at birth, suggesting that the metabolomic profile of a young adult born preterm mirrors that of their perinatal period [9]. The same urine metabolites were also identified as influent in a recent study describing the variation in urine metabolites during the catch-up growth in the first months of life [14]. In this study, the authors found that hippurate and other metabolites were related to an individual’s weight, while citric acid and creatinine were both related to a subject’s weight and height. In the case of citrate, which is part of several pathways regulating carbohydrate, fat and protein metabolism, age-dependent concentrations have been reported in other metabolomic studies. Creatinine is the waste product of the energy muscle metabolism; it is constantly excreted through glomerular filtration, and its concentration in urine and in blood is routinely used as a marker of renal function. Creatinine urinary level appeared to increase with increasing age and body weight, following the increase in muscle metabolism that occurs during childhood and early adult life, with an increase in physical activities [14,15]. The finding that there are specific metabolomic patterns in young adults born preterm that mirror those found in the neonatal period and differ from those found in young adults born at term, confirm that biological samples have unique and distinctive biochemical compositions, which change in response to physiological (body weight, height and age) or pathophysiological stimuli (preterm birth). We hypothesized that an intrauterine environment that is not favorable for optimal embryonic and fetal growth may cause a placental and fetal “reprogramming” with changes in growth patterns and body metabolism that persist, unaltered, over the years [16]. Previous metabolomic studies performed in premature infants have already shown a difference in the levels of amino acids, enzymes and endocrinological markers collected from blood samples in the period immediately after birth (within 24–72 h from birth), showing that children at different stages of prematurity are metabolically distinct [17]. Moreover, it is already known that the adverse environment that preterm infants face during the preconceptual, fetal and postnatal period may have long-lasting effects on their adulthood health [18,19]. Therefore, the “snapshot” produced by the metabolomics provides fingerprinting of the state of health, useful for investigating the body’s metabolomic responses to the disease and external stimuli [20]. Although we believe in the relevance of the link among prenatal environment, fetal growth and adulthood health status in the predictive role of metabolomics in perinatology, the data are too limited to draw definite conclusions regarding the use of metabolomic profiles in clinical practice. Potential confounders (such as dietary intake and hormonal status) should be analyzed in detail and will benefit from studies on a larger number of patients to identify the effect of environmental factors and comorbidities on the metabolomics spectra. In our population, we did not find an association with hypertension or obesity, and we were not able to identify biomarkers for the risk of chronic disease in adulthood. This study has the limitation of including a small number of term control young adults and this may have affected the results for the personal profiles. However, the study population was well defined, with no variability in respect to location, lifestyle and eating habits. Gender and the related hormonal differences may also have influenced these results. This study therefore represents a preliminary phase, and a validation of our results in a new and larger cohort is necessary to check their reproducibility. Looking at the growing global incidence of chronic metabolic diseases, this research contributes to unveil the main routes of reciprocal linking between environmental factors and genetic susceptibility factors. Epigenetic modifications consequent to intrauterine environmental stimuli may persist long after the stimulus has ceased, providing a mechanism to explain the long-term consequences of acute exposures in early life. Metabolomics and ^1^H-NMR allow the analysis of biofluids or tissues to extract latent information and enable sample classification and biomarker identification. Although plasma, serum, amniotic fluid, cord blood or stool can be used for metabolomic analysis, urine samples, due to their non-invasive method of collection, are a very promising tool in the pediatrics and neonatology field. The future goal will be to identify more accurately patients at risk for chronic adult diseases, for which an individual therapeutic approach might be necessary.

## 4. Materials and Methods

An observational case–control monocentric study was carried out at the University Hospital of Siena, in the Neonatology–Pediatrics Unit. The urinary samples were collected from young adults recruited in the research study, “Multidisciplinary long-term follow-up of premature births: AOUS case series 1990–1997”. They were enrolled to take part in the multidisciplinary follow-up study that was conducted at the University Hospital of Siena.

### 4.1. Inclusion and Exclusion Criteria

The study population was enrolled starting from a cohort of young adults born with gestational age (GA) ≤ 33 weeks and/or birth weight ≤ 1500 g, admitted to the Neonatal Intensive Care Unit at the Santa Maria alle Scotte Hospital, in the period between 1 January 1995 and 31 December 1997. Babies born at term in the same study period (years 1995–1997) were selected as controls (for the clinical characteristics of the enrolled population, see Table 1). Subjects suffering from genetic or malformative syndromes, inborn errors of metabolism, severe motor disability and all whose conditions prevented the completion of the performance-expected tests, were excluded from the study. Vegan or vegetarian diet and alcohol use also represented exclusion criteria. The study was conducted in accordance with the ethical principles enshrined in the Helsinki Declaration’s latest revision. Patients eligible for the study were contacted by telephone and informed about the aims and methods of carrying out the study. Adherence to the study was voluntary. Nevertheless, official participation in the study was subject to the signing of an informed written consent, which guaranteed all rights regarding the protection of personal data according to the national law.

### 4.2. Clinical Data Collection

Eligible patients were invited to the Neonatology–Pediatrics Unit, Neurodevelopmental Follow-up Division. For each patient, we drew up a clinical folder, consisting of: a signed copy of the informed consent; data relating to the perinatal age retrospectively collected from the birth medical records (such as gestational age, birth weight, type of delivery, length of hospital stay at birth, complications or problems related to prematurity that came out during hospitalization and diagnosis at discharge); data related to the current state of health of the patient; the anthropometric parameters (including height, weight and body mass index achieved); and the clinical examination.

### 4.3. ^1^H-NMR

Urine samples were collected and shipped in dry ice to the Laboratory of the University of Siena. The samples were then analyzed using the ^1^H-NMR analysis technique. ^1^H-NMR measurements were performed on a Bruker DRX600 MHz Avance Spectrometer with a selective inverse probe equipped with a Z-gradient coil, as previously described [21]. Briefly, spectra were acquired at a constant temperature of 298.0 ± 0.1 K using a 90° pulse. A delay of 10 s was included in the pulse sequence to obtain the relaxation time T1. In fact, the values of T1 (in the range 1.5–2.8 s) of the considered metabolites were such that a delay of 10 s allowed for the full recovery of the longitudinal magnetization after a 90° pulse, as verified by integral values constant for D1 ≥ 5 s. A saturation pulse of 2 s suppressed the water signal during the water resonance. A total of 32 k data points per scan were used and 128 transients were accumulated. Each urine sample was measured after centrifugation occurred, 2000 ppm for 5 min. The pH of the urine samples was checked with a buffer solution (pH 7.4) containing trimethylsilylpropanoic acid (TSP). Samples (550 μL) plus 50 μL of TSP-d4 20 mM solution were measured into the 0.5 mm tube (tube diameter) of the ^1^H-NMR. All ^1^H-NMR spectra were first performed at their physiological pH. This first spectrum was used only to obtain an overview of the metabolites contained. A second spectrum was executed at pH 1.0 ± 0.02 in the same MR tube, with a microelectrode. The chemical shift of ionizable fluids is highly dependent on the pH. At a pH of 1.0, all chemical shift values were reproducible within ± 0.01 ppm. Spectra were aligned to compensate for the shift of the signals of some metabolites, due to small inter-sample pH changes. Then, they were uniformly binned to 0.0025 ppm intervals between 0.5 and 9.5 ppm, excluding the region corresponding to water (4.6–5.2 ppm) and TSP (−0.5–0.5 ppm) signals. Bins were normalized to the total spectral area to compensate for the different dilutions of original urine samples. To identify the most discriminating parts of the spectrum, the results of the classification algorithm were combined with the profiles of the respective loadings. A system of thresholds, defined empirically, then allowed the selection of the characteristic parts of the spectrum of the two groups.

### 4.4. Statistical Analysis

The data were analyzed using the R program (R Core Team (2016). R: a language and environment for statistical computing R Foundation for Statistical Computing, Vienna, Austria. Available online: https://www.r-project.org (accessed on 5 October 2021)). The data of sample characteristics with a normal distribution were evaluated by unpaired t-student test, while categorical data were analyzed by chi-square test. The study was conducted according to the classical metabolomic approach divided into two steps: an unsupervised and a supervised phase [22]. In order to find clustering evidence, a non-supervised technique (PCA) was performed on mean-centered and Pareto-scaled methods data. PCA was also used to detect possible outliers within the dataset. The next supervised step allowed us to model data through different classification systems: RF, support vector machine and gradient boosting machine. These different machine-learning algorithms were used to analyze the differences in the metabolomic profile that were connected to the different gestational age at birth [23,24,25]. As the classification algorithms employed do not provide direct methods for calculating the significance of the variables responsible for classification, alternative methods were used to define which elements could support the performance of the model. To select and identify metabolites that distinguish young adults born preterm from those born at term, a threshold method was used. By varying the threshold of interest, it is possible to look for the metabolites best expressed by the various principal components, and to estimate which are the most important for defining the classification. A threshold method allowed us to combine the rigor of a systematic approach (choice of classification model and identification of the most important principal component) with a more manual approach to test and choose the selection thresholds. It also allowed us to see if metabolites emerge, establishing a significance of the effects.

## 5. Conclusions

Urinary spectra were able to discriminate the metabolomic profiles of young adults born preterm from those born at term, revealing differences similar to those already reported at birth. Urine spectra may provide insight into the peculiar metabolomics of preterm babies that persists into adulthood, paving the way for further research on the pathogenesis and effects of fetal programming on infants’ outcomes. This work is preliminary research that opens the interest of neonatologists to the fingerprinting of prematurity. In-depth knowledge of the metabolomics of preterm babies is very important, not only for a good state of childhood health, but also because we could prevent or intervene, in advance, in situations in which neonatal development is at risk to become poor. This would represent, in the clinical setting, a relatively inexpensive and non-invasive screening tool for some early-life pathologies that involve pathophysiological alterations of the metabolism itself.

## Figures and Tables

**Table 1:** Perinatal and actual data in case and control groups.

Variables	Cases (*n* = 49)	Controls (*n* = 18)	*p*-Value
Maternal age (years), mean (SD)	31.19 (4.72)	31.15 (4.04)	Ns
Gestational age (weeks), mean (SD)	30.25 (2.72)	38.52 (1.44)	<0.05
Birth weight (grams), mean (SD)	1131.91 (118.15)	3120.43 (261.02)	<0.05
Male gender, n (%)	31 (63.26)	12 (66.6)	Ns
Apgar score at 1 min, median (IR)	5 (1–10)	9 (8–10)	<0.05
Apgar score at 5 min, median (IR)	8 (1–10)	10 (10–10)	<0.05
Neonatal resuscitation, n (%)	43 (87.7)	-	-
Intraventricular hemorrhage, n (%)	16 (32.6)	-	-
Hospital stay (months), mean (SD)	2.15 (1.11)	-	-
Age at assessment (years), mean (SD)	21.68 (2.42)	20.95 (2.55)	Ns
Caucasian population, n (%)	47 (95.9)	18 (100)	Ns
Same region of residency, n (%)	48 (97.9)	16 (88.8)	Ns
Actual mean systolic/diastolic blood pressure values (mmHg)	105/73	108/75	Ns
Actual body mass index < 18.5, n (%)	11 (22.4)	4 (22.2)	Ns
Actual body mass index 18.5–25, n (%)	34 (69.4)	13 (72)	Ns
Actual body mass index > 25, n (%)	4 (8.1)	1 (5.5)	Ns
Sport, n (%)	16 (32.6)	7 (38.9)	Ns

Ns: non significative.

**Table 2:** Performance of the classification algorithms, obtained using scaled spectral data as input.

	Accuracy	F1 Measure	False Positive Rate	False Negative Rate	True Positive Rate	True Negative Rate
RF	0.7	0.82	0.94	0.06	0.94	0.06
GBM	0.72	0.82	0.72	0.12	0.88	0.28
SVM	0.73	0.84	1	0	1	0

RF: Random Forest; GBM: gradient boosting machine; SVM: support vector machine.

## Data Availability

Because of the participant consent obtained as part of the recruitment process, it is not possible to make these data publicly available. The data resented in this study are available on request from the corresponding author.
